# Supplementary material for: Improved clearing method contributes to deep imaging of plant organs
Source: Commun Biol. 2022 Jan 10;5:12. doi: 10.1038/s42003-021-02955-9 (PMC8748589; doi:10.1038/s42003-021-02955-9)
Supplement: Supplementary file 1 — Editorial Summary [file 42003_2021_2955_MOESM1_ESM.docx]

**Sakamoto et al. demonstrate an improved optical clearing method, iTOMEI, for plant imaging. The new method can achieve fast clearing and effective removal of autofluorescence signals, and at the same time preserve signals from desired fluorescence proteins.**
