# Supplementary material for: Improved clearing method contributes to deep imaging of plant organs
Source: Commun Biol. 2022 Jan 10;5:12. doi: 10.1038/s42003-021-02955-9 (PMC8748589; doi:10.1038/s42003-021-02955-9)
Supplement: Supplementary file 3 — Supplementary Information [file 42003_2021_2955_MOESM3_ESM.pdf]

Supplementary information

"Improved clearing method contributes to deep imaging of plant organs"

Yuki Sakamoto[1,2], Anna Ishimoto[3], Yuuki Sakai[4], Moeko Sato[5], Ryuichi Nishihama[3,6], Konami Abe[3], Yoshitake Sano[1,3], Teiichi Furuichi[1,3], Hiroyuki Tsuji[5], Takayuki Kohchi[6], and Sachihiro Matsunaga[1,3,7]

[1] Imaging Frontier Center, Organization for Research Advancement, Tokyo University of Science, 2641 Yamazaki, Noda, Chiba 278-8510, Japan

[2] Department of Biological Sciences, Graduate School of Science, Osaka University, Machikaneyama-cho 1-1, Toyonaka, Osaka, 560-0043, Japan

[3] Department of Applied Biological Science, Faculty of Science and Technology, Tokyo University of Science, 2641 Yamazaki, Noda, Chiba 278-8510, Japan

[4] Department of Biology, Graduate School of Science, Kobe University, Kobe, 657-8501, Japan

[5] Kihara Institute for Biological Research, Yokohama City University, Maioka 641-12, Totsuka, Yokohama, 244-0813, Japan

[6] Graduate School of Biostudies, Kyoto University, Kyoto 606-8502, Japan

[7] Department of Integrated Biosciences, Graduate School of Frontier Sciences, The University of Tokyo, 5-1-5 Kashiwanoha, Kashiwa, Chiba 277-8562, Japan

This file includes Supplementary Fig. 1-5 and Supplementary Table 1.

Supplementary Fig. 1

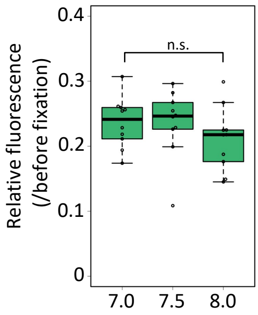

Supplementary Fig. 1 Effects of different pH levels of fixation buffers on GFP

Relative fluorescence intensity quantified from fluorescence images of plants expressing GFP before and after fixation independently in 2% FA buffer at pH 7.0, 7.5, and 8.0 for 1 h (n = 10). Statistical significance was evaluated using a two-sided Welch's t-test.

Supplementary Fig. 2

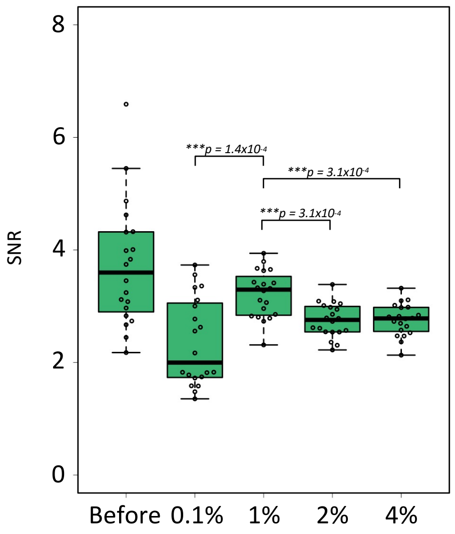

Supplementary Fig. 2 Signal-to-noise ratios of the samples fixed with different FA concentrations
The seedlings expressing PCNA-GFP were fixed independently with 0.1%, 1%, 2%, and 4% FA. The fluorescence signals were detected in the nucleoplasm, and the noise was detected in the cytoplasm. A value of noise over signal was calculated as the signal-to-noise ratio (n = 20). Statistical significance was evaluated using a two-sided Welch's t-test.

Supplementary Fig. 3

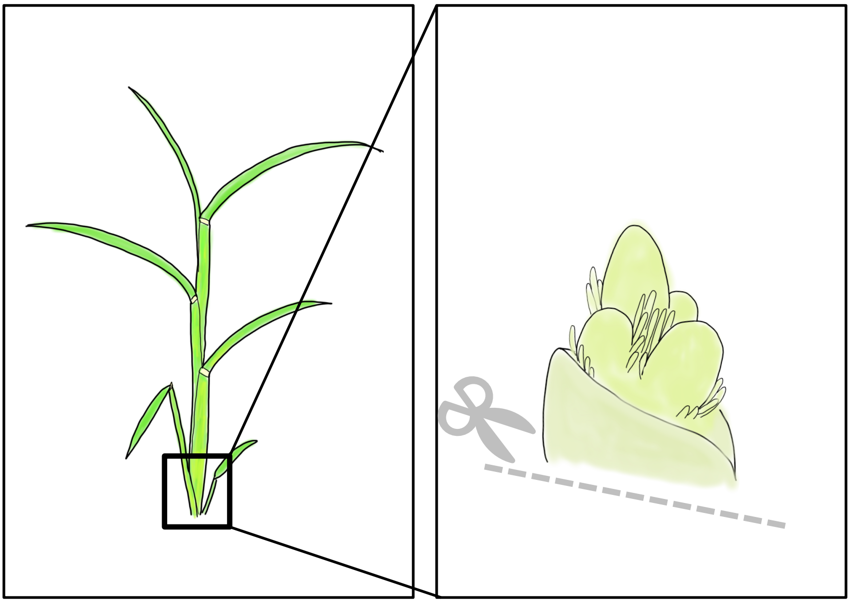

Supplementary Fig. 3 Schematic diagram of the dissection of SAM from rice
Before fixation, we dissected the SAM from a rice plant.

Supplementary Fig. 4

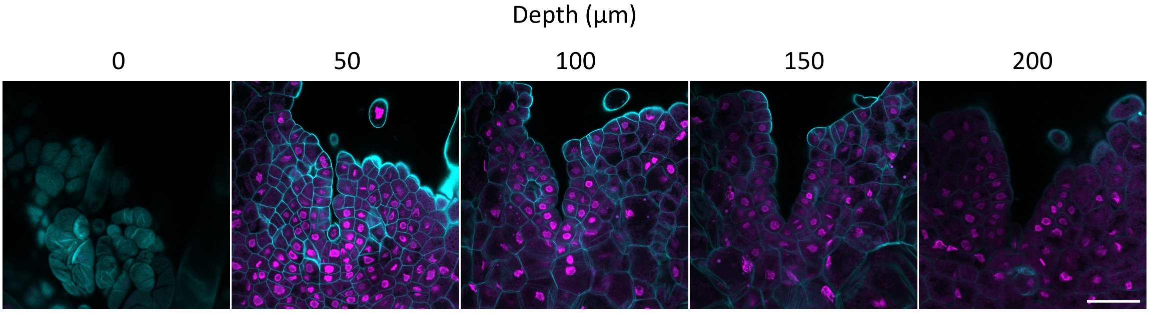

Supplementary Fig. 4 Deep imaging of a transparent apical region of *M. polymorpha* using iTOMEI

A thallus of a 3-day-old gemmaling expressing H2B–tdTomato (magenta) stained with Calcofluor White (cyan) and treated with iTOMEI. Confocal optical sections were captured from the ventral surface. A scale bar = 50 µm.

Supplementary Fig. 5

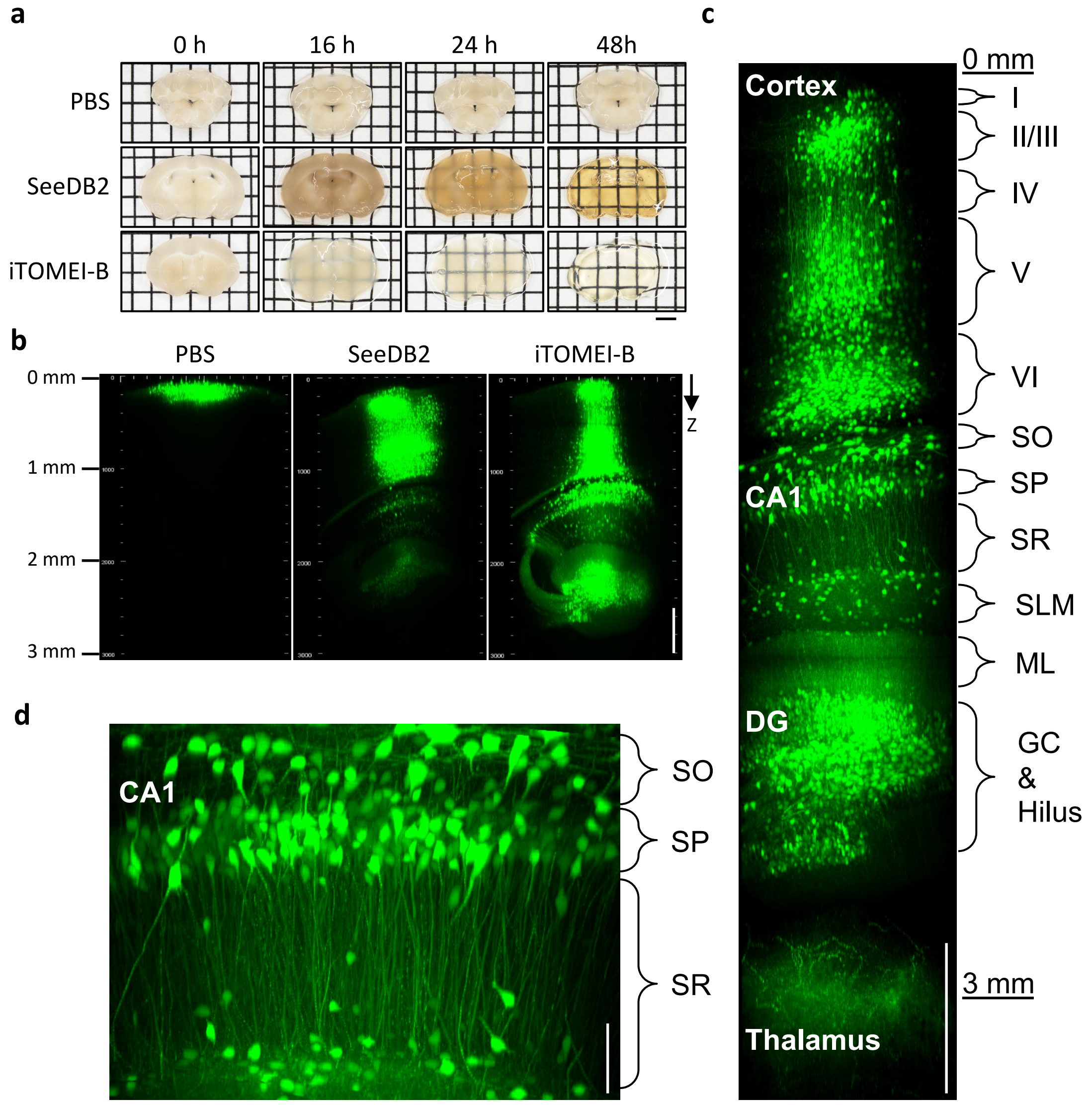

Supplementary Fig. 5 Deep imaging of transparent mouse brain using iTOMEI-B

a Mouse brains treated independently with PBS buffer, SeeDB2, and iTOMEI-B for 0, 16, 24, and 48 h. **b** Three-dimensional reconstruction images of mouse brains expressing GFP after treatment with various solutions for 48 h. Images were captured from the cortical surface of the hemi-brain under the same imaging conditions using a two-photon excitation microscope. **c** Three-dimensional reconstruction of neurons expressing GFP in the transparent brain using iTOMEI-B. **d** Enlarged image of CA1 after iTOMEI-B treatment. The depth from the cortex is indicated on the right. Abbreviations: CA, cornu ammonis; DG, dentate gyrus; SO, stratum oriens; SP, stratum pyramidal; SR, stratum radiatum; SLM, stratum lacunosum-moleculare; ML, molecular layer; GC, granule cell layer, I–VI; cortical cell layers I–VI. Scale bars = 2 mm (a), 500 µm (b, c), and 100 µm (d).

Supplementary Table 1

Number	Chemical name
#1	Sodium Lauryl Sulfate
#2	Lithium Dodecyl Sulfate
#3	Sodium Deoxycholate
#4	Sodium Cholate
#5	Sodium N-Lauroylsarcosinate
#6	*n*-Octyl Sulfobetaine
#7	Caprylyl Sulfobetaine
#8	Lauryl Sulfobetaine
#9	Myristyl Sulfobetaine
#10	Palmityl Sulfobetaine
#11	Tween 20
#12	Tween 40
#13	Tween 80
#14	Polyethylene Glycol Monododecyl Ether
#15	Polyethylene Glycol Monocetyl Ether
#16	Polyethylene Glycol Mono-4-octylphenyl Ether
#17	*n*-Octyl β-D-Glucopyranoside
#18	TritonX-100
#19	NP-40
