# Supplementary material for: Improved clearing method contributes to deep imaging of plant organs
Source: Commun Biol. 2022 Jan 10;5:12. doi: 10.1038/s42003-021-02955-9 (PMC8748589; doi:10.1038/s42003-021-02955-9)
Supplement: Supplementary file 6 — Description of Additional Supplementary Files [file 42003_2021_2955_MOESM6_ESM.pdf]

Description of Additional Supplementary Files

File name: Supplementary Data

Description: Our source data for graphs and charts are uploaded as Supplementary Data. The captions for each Supplementary Data are described below.

Supplementary Data 1. Raw data of each graph in Fig. 1a, b and c.

Supplementary Data 2. Raw data of each graph in Fig. 2b and c.

Supplementary Data 3. Raw data of each graph in Fig. 3a and b.

Supplementary Data 4. Raw data of each graph in Fig. 4b, d and e.

Supplementary Data 5. Raw data of each graph in Fig. 6d.
